# Association between LCE gene polymorphisms and psoriasis vulgaris among Mongolians from Inner Mongolia

**DOI:** 10.1007/s00403-018-1813-0

**Published:** 2018-02-03

**Authors:** Li Sun, Yuting Cao, Nagonbilig He, Jianwen Han, Rong Hai, Sarnai Arlud, Baoyindeligeer He, Wurina Wu, Lizhong Li, Xiulan Su, Hongwei Cui, Wenchao Zhao, Buheqiqige Chao, Dandan Liu, Zhiqiang Sun, Yanping Huang

**Affiliations:** 1 Department of Dermatology, Affiliated Hospital of Inner Mongolia Medical University, Hohhot, China; 2 Department of Dermatology, Inner Mongolia Maternal and Child Health Care Hospital, Hohhot, China; 3 Department of Psychosomatic Medicine, Inner Mongolia International Mongolian Hospital, Hohhot, China; 4 Cadre Health Center of Inner Mongolia People’s Hospital, Hohhot, China; 5 Department of Dermatology, Inner Mongolia International Mongolian Hospital, Hohhot, China; 6 Maternal and Child Health Care and Family Planning Service Center, Qingshui River, Hohhot, Inner Mongolia China; 7 Clinical Medical Research Center, Affiliated Hospital of Inner Mongolia Medical University, Hohhot, China; 8 Blood Center of Inner Mongolia, Hohhot, China

**Keywords:** Mongolians, Inner Mongolia, Psoriasis vulgaris, LCE gene, Single nucleotide polymorphisms

## Abstract

The late cornified envelope (LCE) gene cluster is located on chromosome 1q21, including LCE1–LCE6. Several single nucleotide polymorphisms (SNPs) in the LCE cluster were associated with susceptibility to psoriasis in Chinese population. However, there is no report on the relationship in ethnic minority areas in China. This study aimed to investigate the association between the gene polymorphisms of LCE1B, LCE1C, LCE3A, LCE3D and psoriasis vulgaris among Mongolians from Inner Mongolia. Totally, 305 Mongolians with psoriasis vulgaris (PsV) and 383 healthy controls were enrolled in the study from 2006 to 2015. 7 SNPs including rs6701216, rs4112788, rs12023196, rs512208, rs4845454, rs4085613 and rs1886734, were selected for genotyping with ligase detection reaction (LDR). Statistical analysis was performed for comparisons of allele frequencies and genotype frequencies between the patient group and the control group. In this study, excluding rs4085613 and rs1886734, differences were detected in the allele frequencies of other 5 SNPs between the patients and controls. Genotype analysis showed that under the recessive inheritance model, the genotype frequencies of rs4845454, rs4112788 differed between the patients and controls (all *p* < 0.00 5).Under the dominant and the recessive model, the genotype frequencies of rs6701216, rs12023196 and rs512208 significantly differed between the patients and controls. The LD analysis showed that strong LD existed between rs6701216 and rs12023196, rs4845454 and rs4085613, rs4845454 and rs1886734, and rs4085613 and rs1886734. The SNPs rs6701216, rs4112788, rs12023196, rs512208 and rs4845454 in the LCE gene were associated with psoriasis vulgaris among Mongolians from Inner Mongolia.

**Electronic supplementary material:**

The online version of this article (10.1007/s00403-018-1813-0) contains supplementary material, which is available to authorized users.

## Introduction

Psoriasis is a common, recurrent, chronic, inflammatory skin disease with a prevalence of 0.47% in Chinese [12, 16]. It is characterized by high epidermis proliferation, abnormal keratinocytes differentiation and inflammatory reaction [16]. The pathogenesis of this complicated disease remains unclear. Recent studies suggest that genetic factors have relatively important influence on the pathogenesis of psoriasis. Since the nineties of the last century, with the development of molecular biology technology, the research on psoriasis susceptibility genes has shown promising results. Notably, completion of the human genome project (HGP) has resulted in landmark progress in the study of genetic diseases such as psoriasis. But psoriasis is a complex genetic disease, which is affected by genetic factors, geographical, ethnic and environmental stimulation. Therefore, to explore the pathogenesis and find the susceptible genes of psoriasis, it is also necessary to investigate different populations worldwide. Mongolians are an important group in north China. As compared to other nationalities, Mongolians have unique genetic background, living environment and living habits. Therefore, study on susceptibility genes of psoriasis in Mongolians will also provide valuable data on the genetics of psoriasis and more theoretical basis for the diagnosis and treatment of psoriasis at the gene level in the future.

Since 2008, a large number of susceptible genes of psoriasis were found [2, 6]. The LCE gene is located in the epidermal differentiation complex region of chromosome 1q21.3 [4], which can be further divided into six subgroups [17]. Among them, LCE3 gene cluster contains LCE3A, LCE3B, LCE3C, LCE3D and LCE3E, which have different structure and function [5]. The association between LCE and psoriasis was confirmed in different ethnic groups [1, 8, 15]. In 2008, new susceptibility loci for psoriasis-LCE1C (rs6701216) was found through GWAS in Europeans and Americans [9]. In 2009, LCE3D (rs4112788, rs4085613) and LCE3A (rs4845454, rs1886734) [8] was reported to be associated with psoriasis in Han and Uygur. In addition, this research also confirmed that LCE1B (rs12023196) may be associated with susceptibility of psoriasis in Chinese Han population. Huayang Tang [9] et al. confirmed that rs512208 in LCE3D was significantly associated with psoriasis in Chinese. In our study, seven single nucleotide polymorphisms (SNPs) in the LCE region, which are mentioned above, were chosen to explore their relationship with the Mongolian psoriasis vulgaris in Inner Mongolia of China.

## Materials and methods

### Materials

#### Subjects and controls

From January 2006 to December 2015, a total of 305 psoriasis vulgaris and 383 healthy controls were enrolled into the study. All patients and controls were Mongolians. The patients were recruited from the Dermatology department of the Affiliated Hospital of Inner Mongolia Medical University, in accordance with the diagnostic criteria of psoriasis vulgaris after being diagnosed by four clinical dermatologists through clinical or pathological methods. All controls were healthy people who had undergone physical examination in the Affiliated Hospital of Inner Mongolia Medical University. The inclusion criteria for controls were: (1) no autoimmune and systemic diseases, (2) no history of psoriasis, (3) no family history of psoriasis in one, two, three degree relatives. All controls were gender-matched with the psoriasis patients. More than three generations of the subjects and controls in our study had been living in Inner Mongolia and were not related to each other.

All patients and controls accepted a rigorous questionnaire survey and follow-up with informed consent. The study was approved by the ethics committee of the Affiliated Hospital of Inner Mongolia Medical University (Ethical approval number: 2007-001). The investigations were conducted according to the Declaration of Helsinki principles.

#### Selection of SNPs

Based on previous reports, 7 SNPs including rs6701216, rs4112788, rs12023196, rs512208, rs4845454, rs4085613 and rs1886734 in the LCE region were selected in this study^3,5,7,8,13, 14, 15^.

### Methods

#### Genomic DNA extraction

Two milliliters of venous blood samples were collected from each subject after signing informed consent. Edtap dipotassium ethylene diamine tetraacetate (EDTAK2) was added into the blood samples as anticoagulant. All samples were stored at − 80 °C. AxyPrep-96 Whole blood genomic DNA kit from AXYGEN (33210 Central Avenue Union City, California, 94587, USA) was used to extract genomic DNA from patients and controls. The absorbance at 260 and 280 nm were detected by spectrophotometer. The concentration of DNA was calculated. The integrity of DNA was detected by 0.8% agarose gel electrophoresis.

#### Synthesis of primers and probes

Primer 3 online (Version 0.4.0) (http://frodo.wi.mit.edu/) and oligo (Version 6.31) (Molecular biology insights Inc, USA) softwares were used to design LDR probes and specific primers. Two primers were designed for each SNP locus. We tried our best to ensure that all SNP loci were in the same T value. Polymerase chain reaction (PCR) primer sequences are shown in Table 1. The upstream and downstream probes of the ligase detection reaction (LDR) were designed according to the design principle of LDR probe [3, 7]. The 5′end of the upstream probe was modified by phosphorylation. The LDR probe sequence is shown in the animation (Online Resource).

**Table 1:** PCR primer sequences

SNP	Upstream (5′–3′)	Downstream (3′–5′)	PCR product length (bp)
rs4845454	GGGTCACAAATTCAGAAAGG	TGACCACAGCTCCAATCAAC	82
rs1886734	CCATAAGGAGCTTGCCCATC	CTGGTACACTTAAGACATGC	98
rs6701216	ACCAGCCTAGAGCCAGGGCA	CACAGGCTCCCTTTGTTAAG	92
rs4085613	ACTCCTTGAGAGCCCTTTTC	GAAAACGTCAAACTGCCTAT	99
rs12023196	AGGGCCAAAACTTCAAAGCT	CTGCTTCAGTACCCAGGGAA	375
rs512208	GGCCGCTGGTCTTAGAGACA	CAGGATCCAGGTCAGCAGCAGCCT	378
rs4112788	CCCAGTCGTAGGAGGAGCTA	TCTGCCACTATGCCAAGCTA	497

#### Multiplex PCR

PCR reaction system was 20 µl, which consisted of the following components: 1 µl templates, 2 µl buffer, 0.6 µl Mg^2+^, 2 µl dNTP, 0.2 µl TaqDNA polymerases, 2 µl primer mix and 12.2 µl H_2_O. The cycling conditions included initial denaturation at 95 °C for 15 min, 35 cycles of three steps (denaturation at 94 °C for 30 s, annealing at 56 °C for 1 min and extension at 65 °C for 30 s), followed by a final extension at 65 °C for 10 min.

#### Multiplex LDR

The ligation reaction was performed in a final volume of 10 µl containing 1 µl buffer, 1 µl probe mixture, 0.05 µl Taq DNA ligase, 4 µl PCR product and 3.95 µl H_2_O. Probe mixture concentration was 2 pmol/µl. The cycling conditions included denaturation at 95 °C for 2 min, 40 cycles of two steps (annealing at 94 °C for 15 s and extension at 50 °C for 25 s).

#### Sequencing and genotyping

The reaction products were sequenced and analyzed using PRISM 3730 sequencer. Finally, data were analyzed using GeneMapper software. The sequencing was completed by the Shanghai Yihe application of Biotechnology Co. Ltd.

### Statistical analysis

Statistical analyses were performed using PLINK 1.07 software (http://pngu.mgh.harvard.edu/purcell/plink/) [3]. All allele and genotype frequencies of 7 SNPs in the case and control groups were calculated, and the Hardy–Weinberg genetic equilibrium analysis was performed. Chi-square test (*χ*^2^) was used to compare the allele and genotype frequencies. Differences in allele frequencies were quantified by OR and 95% CI using SPSS for Windows software (version 17.0; SPSS, Chicago, IL, USA). Linkage disequilibrium between the 7 SNPs was analyzed, and the values of *R*^2^ and D were calculated. All *P* values were two-sided, and *p* < 0.05 was considered to be statistically significant, *p* < 0.007 (0.05/7) was considered to achieve the Bonferroni multiple test correction level.

## Results

### Sample and SNP quality control

A total of 305 psoriasis vulgaris (mean age 40.20 ± 15.67, 171 males and 134 females) patients and 383 healthy controls (mean age 28.71 ± 12.36, 181 males and 202 females) were enrolled into the study. There was no significant difference in gender and age distribution between the patient and control groups (*p* > 0.05). The distribution of the 7 SNPs was in accordance with the Hardy–Weinberg equilibrium in both groups (*p* > 0.1).

**Table 2:** Allele frequency for PsV and controls

SNP	Allele	Number (Allele frequency %)		*χ* ^2^	*p*	OR (95% CI)
		Control *N* = 383	Patient *N* = 305			
rs4845454	C	314 (40.99)	283 (46.39)	4.033	0.045	1.246 (1.005–1.544)
rs6701216	T	240 (31.33)	257 (42.13)	17.165	< 0.001	1.596 (1.278–1.992)
rs1886734	T	320 (41.78)	285 (46.72)	3.372	0.066	1.222 (0.986–1.514)
rs4085613	A	329 (42.95)	294 (48.20)	3.772	0.052	1.236 (0.998–1.530)
rs12023196	C	255 (33.29)	282 (46.23)	23.894	< 0.001	1.723 (1.384–2.144)
rs512208	T	220 (28.72)	276 (45.25)	40.266	< 0.001	2.051 (1.640–2.564)
rs4112788	T	296 (38.64)	295 (48.36)	13.089	< 0.001	1.487 (1.199–1.844)

The difference of allele frequency of the other 3 SNPs between the PsV and the controls did not reach the Bonferroni correction level

*OR* odds ratio, *CI* confidence interval, *P* asymptotic *p* value for the epistatic effect between the patient and control

### Allele frequency comparison (Table 2)

**Fig. 1:**
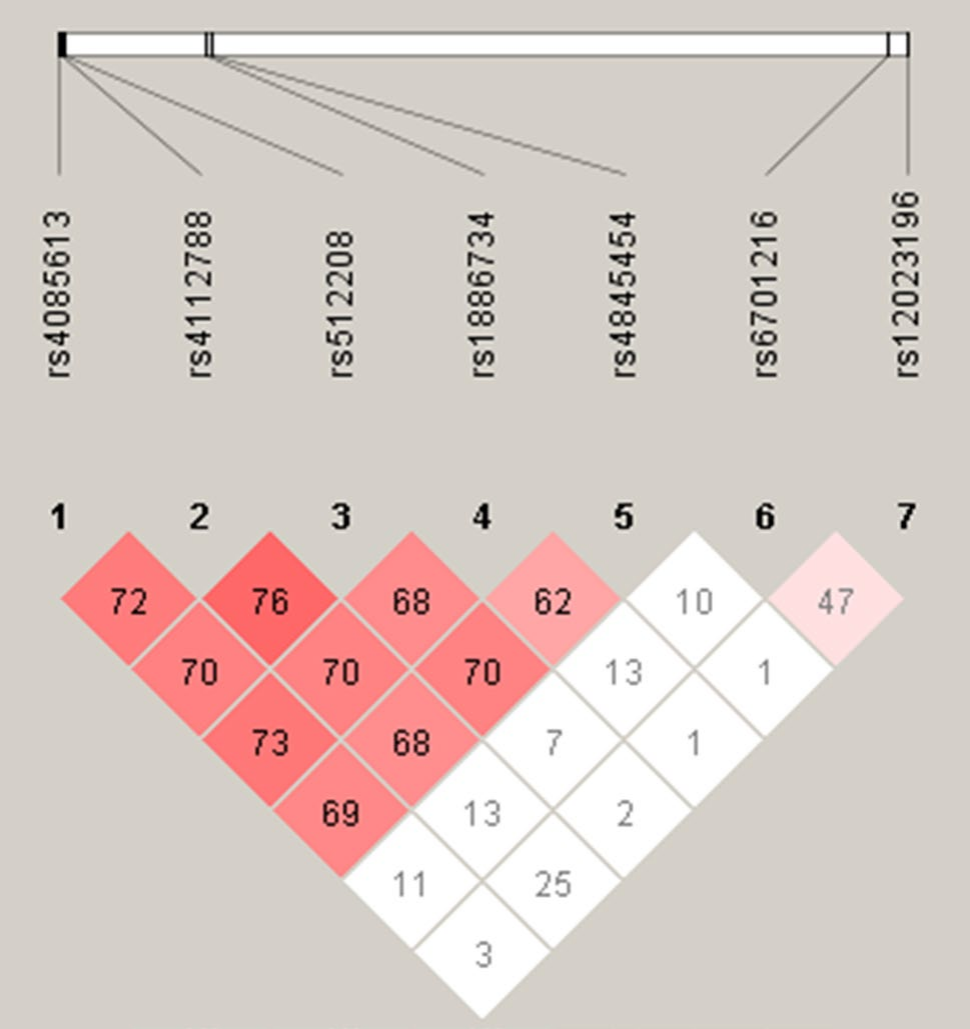
Linkage disequilibrium (LD) map across the 7 SNPs. The measure of LD (*D*′) is shown graphically between all possible pairs of SNPs in terms of the shade of the color (*D*′), where white represents very low *D*′ and dark represents very high *D*′. The numbers in squares are D’values

The linkage disequilibrium (LD) analysis between every 2 SNPs showed strong LD between rs6701216 and rs12023196 (*R*^2^ = 0.720, *D* = 0.903), rs4845454 and rs4085613 (*R*^2^ = 0.681, *D* = 0.857), rs4845454 and rs1886734 (*R*^2^ = 0.705, *D* = 0.849), and rs4085613 and rs1886734 (*R*^2^ = 0.680, *D* = 0.847) (Fig. 1).

### Genotype frequency comparison

Genotype analysis showed that under the recessive inheritance model, the genotype frequencies of rs4845454, rs4112788 differed between the patients and controls, all of which can reach the level of Bonferroni correction(all *p* < 0.00 7).Under the dominant and the recessive model, the genotype frequencies of rs6701216, rs12023196 and rs512208 significantly differed between the patients and controls. (Table 3). In addition, we also used the logical regression method to analyze the additive models of the 7 SNPs. The results showed that under the additive model, the *P* values of rs6701216, rs12023196, rs512208 and rs4112788 were less than 0.05, all of which can reach the level of Bonferroni correction(all *p* < 0.00 7), indicating that these 4 SNPs were significantly correlated with psoriasis (Table 4).

**Table 3:** Genotype analysis of 7 SNPs

SNP	Genetic model	Group	Genotype distribution	χ^2^	P	OR (95% CI)
rs6701216	Genotype	Patient	107/139/59			
	CC/CT/TT	Control	179/168/36			
	Dominant	Patient	198/107	9.494	0.002	1.624(1.192–2.212)
	(TT + CT/CC)	Control	204/179			
	Recessive	Patient	59/246	14.109	0.000	2.312(1.481–3.609)
	(TT/CT + CC)	Control	36/347			
rs4112788	Genotype	Patient	98/119/88			
	(CC/CT/TT)	Control	148/174/61			
	Dominant	Patient	207/98	3.134	0.077	1.330 (0.970–1.825)
	(TT + CT/CC)	Control	235/148			
	Recessive	Patient	88/217	16.719	0.000	2.141(1.480–3.096)
	(TT/CT + CC)	Control	61/322			
rs12023196	Genotype	Patient	79/124/102			
	(CC/CT/TT)	Control	42/171/170			
	Dominant	Patient	203/102	8.507	0.004	1.588(1.163–2.170)
	(CC + CT/TT)	Control	213/170			
	Recessive	Patient	79/226	26.131	0.000	2.838(1.883–4.278)
	(CC/CT + TT)	Control	42/341			
rs512208	Genotype	Patient	104/126/75			
	(GG/GT/TT)	Control	193/160/30			
	Dominant	Patient	201/104	18.373	0.000	1.963(1.440–2.677)
	(TT + GT/GG)	Control	190/193			
	Recessive	Patient	75/230	36.867	0.000	3.837(2.435–6.047)
	(TT/GT + GG)	Control	30/353			
rs4845454	Genotype	Patient	100/127/78			
	(TT/CT/CC)	Control	129/194/60			
	Dominant	Patient	205/100	0.061	0.805	1.041(0.756–1.433)
	(CC + CT/TT)	Control	254/129			
	Recessive	Patient	78/227	10.395	0.001	1.850 (1.269–2.697)
	(CC/CT + TT)	Control	60/323			
rs4085613	Genotype	Patient	98/120/87			
	(CC/AC/AA)	Control	124/189/70			
	Dominant	Patient	207/98	0.005	0.946	1.011(0.733–1.395)
	(AA + AC/CC)	Control	259/124			
	Recessive	Patient	87/218	10.124	0.001	1.784(1.246–2.555)
	(AA/AC + CC)	Control	70/313			
rs1886734	Genotype	Patient	79/127/99			
	(TT/GT/GG)	Control	63/194/126			
	Dominant	Patient	206/99	0.015	0.903	1.020(0.740–1.406)
	(TT + GT/GG)	Control	257/126			
	Recessive	Patient	79/226	9.262	0.002	1.776(1.224–2.576)
	(TT/GT + GG)	Control	63/320			

*OR* odds ratio, *CI* confidence interval, *P* asymptotic *p* value for the epistatic effect between the patient and control

**Table 4:** Logistic regression analysis of 7 SNPs

SNP	CHR	BP	Alt Allele	Model	NMISS	OR	SE	L95	U95	STAT	*P* value	FDR_BH adjusted
rs1886734	1	152,591,142	T	Additive	688	1.21	0.1069	0.9816	1.492	1.787	0.074	0.074
rs4085613	1	152,550,018	A	Additive	688	1.214	0.1046	0.989	1.49	1.854	0.0637	0.074
rs6701216	1	152,778,526	T	Additive	688	1.58	0.1133	1.266	1.973	4.04	0.00005338	0.0001246
rs12023196	1	152,783,724	C	Additive	688	1.652	0.1088	1.335	2.044	4.613	0.000003974	0.00001391
rs4845454	1	152,592,184	C	Additive	688	1.233	0.1073	0.9996	1.522	1.956	0.05046	0.07064
rs512208	1	152,552,285	T	Additive	688	1.954	0.1128	1.566	2.437	5.936	2.915E−09	2.041E−08
rs4112788	1	152,551,276	T	Additive	688	1.424	0.1044	1.16	1.747	3.384	0.0007145	0.00125

*CHR* chromosome, *BP* mutation position, *Alt Allele* another allele of a loci, *NMISS* data analysis, *OR* odds ratio, *SE* standard deviation, *L95,U95* 95% confidence interval of logical regression, *STAT* statistics of T, *FDR_BH adjusted* FDR correction

## Discussion

In this study, we replicated the previously reported association of 7 SNPs with Mongolian psoriasis in Inner Mongolia. Ying Liu et al. confirmed that the expression frequency of LCE1C (rs6701216) in Caucasians with psoriasis was significantly higher than that in the control group, and the allele T was associated with the risk of psoriasis [9], which is consistent with our results. 4 SNP loci including rs4112788, rs4845454, rs1886734, and rs4085613 were found to be significantly associated with psoriasis in Chinese Han and Uygur by Zhang Xuejun [8]. We further detected these 4 susceptibility loci in 305 Mongolians with psoriasis vulgaris in Inner Mongolia of China. However, differences were detected in the allele frequencies of only 2 SNPs (rs4112788, rs4845454) between the patients and controls. The other 2 SNPs (rs4085613、rs1886734) of them were confirmed having no significant difference between patients and controls in our study. Besides, Zhang Xuejun [8] showed that the correlation between SNP rs6701216 and Chinese psoriasis patients was very weak (p = 0.02), which is very different from our study. We analyzed that the results may be affected by race and environmental factors, which need to further expand the sample for validation. We used the ligase detection reaction for rs12023196 genotyping in 305 Mongolians with psoriasis vulgaris and 383 normal controls, which showed that the allele frequencies in the case and control groups had significant differences (*p* < 0.001, OR = 1.723, 95% CI = 1.384–2.144). Besides, the allele T of rs12023196 was associated with the risk of psoriasis. Prior to this, Zhang Xuejun et al. had also arrived at the same conclusion in a genetic study of 1103 Chinese Han patients with psoriasis. In this study, we confirmed that LCE3D (rs512208) was significantly associated with Mongolian psoriasis in Inner Mongolia, which was consistent with the findings of Huayang Tang [9]. The results of this study showed that the 5 SNPs in LCE gene were related to Mongolian psoriasis in Inner Mongolia. The linkage disequilibrium (LD) analysis showed strong LD between rs6701216 and rs12023196, rs4845454 and rs4085613, rs4845454 and rs1886734, and rs4085613 and rs1886734. Genotype analysis showed that the allele C of rs4845454, allele A of rs4085613 and allele T of rs1886734 may be recessive. Besides, the analysis of the additive models of these 7 loci can further confirm that rs6701216, rs12023196, rs512208 and rs4112788 were significantly correlated with psoriasis vulgaris among Mongolians from Inner Mongolia.

In conclusion, this study confirmed the association of LCE gene polymorphism with Mongolian psoriasis in Inner Mongolia through genetic methods, and further proved that the LCE gene may play an important role in the pathogenesis of psoriasis, providing new insights into the genetics of psoriasis. But there are some limitations in this research. First, this study mainly focused on the association analysis of single gene polymorphisms, and did not explore the complicated interaction between LCE gene polymorphisms and other genes or clarify whether the association between genetic variation and disease susceptibility was affected by other genes. Second, how LCE polymorphisms affect its gene function and immune system function needs more in-depth study in the future.

## Electronic supplementary material

Below is the link to the electronic supplementary material.

Supplementary material 1 (PDF 81 KB)
