# Supplementary material for: Association between LCE gene polymorphisms and psoriasis vulgaris among Mongolians from Inner Mongolia
Source: Arch Dermatol Res. 2018 Feb 3;310(4):321–7. doi: 10.1007/s00403-018-1813-0 (PMC5915497; doi:10.1007/s00403-018-1813-0)
Supplement: Supplementary file 1 — Supplementary material 1 (PDF 81 KB) [file 403_2018_1813_MOESM1_ESM.pdf]

Supplementary data. LDR probe sequence

Probe name	Probe sequence (5’- 3’)	Product length
rs6701216_modify	P-ATCCCCTCTATGCACCAAAAATACTTTTTTTTTTTTTTTTTT TTTTTTTTTTTTTTTTTTTTTTTTTTTTTTTTTTTTT-FAM	
rs6701216_C	TTTTTTTTTTTTTTTTTTTTTTTTTTTTTTTTTTTTTTTTTTTTTT TTTTCTAGAGCCAGGGCACCTGAGTTCAG	154
rs6701216_T	TTTTTTTTTTTTTTTTTTTTTTTTTTTTTTTTTTTTTTTTTTTTTT TTTTTTCTAGAGCCAGGGCACCTGAGTTCAA	156
rs4112788_modify	P-TCTTTTAATTGTAGTCTAAAGGTTTTTTTTTTTTTTTTTT TTTTTTTTTTTTTTTTTTTTTTTTTTTTTTTTTTTTT-FAM	
rs4112788_C	TTTTTTTTTTTTTTTTTTTTTTTTTTTTTTTTTTTTTTTTTTTTTT TTTTTTTTTTTTCTGAGGCCATGTTTTTCTTTACCCG	162
rs4112788_T	TTTTTTTTTTTTTTTTTTTTTTTTTTTTTTTTTTTTTTTTTTTTTT TTTTTTTTTTTTTTCTGAGGCCATGTTTTTCTTTACCCA	164
rs12023196_modify	P-TAGGTCCCGTGACAGGTGGTAACTGTTTTTTTTTTTTTTTT TTTTTTTTTTTTTTTTTTTTTTTTTTTTTTTTTTTTTT-FAM	
rs12023196_C	TTTTTTTTTTTTTTTTTTTTTTTTTTTTTTTTTTTTTTTTTTTTTT TTTTTTTTTTTTTTTTGAGGACTTGCCTCCTGACACCCACG	166

rs12023196_T	TTTTTTTTTTTTTTTTTTTTTTTTTTTTTTTTTTTTTTTTTTTTTT<br>TTTTTTTTTTTTTTTTTTGAGGACTTGCCTCCTGACACCCACA	168
rs512208_modify	P-CCCCAGAGCTTGGGGCACAGCCAGATTTTTTTTTTTTTTTT<br>TTTTTTTTTTTTTTTTTTTTTTTTTTTTTTTTTTTTTT-FAM	
rs512208_G	TTTTTTTTTTTTTTTTTTTTTTTTTTTTTTTTTTTTTTTTTTTTTT<br>TTTTTTTTTTTTTTTTTTTTCCGCCCTCGGAGCTAGGGCCACA<br>GC	170
rs512208_T	TTTTTTTTTTTTTTTTTTTTTTTTTTTTTTTTTTTTTTTTTTTTTT<br>TTTTTTTTTTTTTTTTTTTTTTCCGCCCTCGGAGCTAGGGCCA<br>CAGA	172
rs4845454_modify	P-CCCTGCTGAGTCCTTTCTGAATTTGTTTTTTTTTTTTTTTTT<br>TTTTTTTTTTTTTTTTTTTTTTTTTTTTTTTTTTTTT-FAM	
rs4845454_C	TTTTTTTTTTTTTTTTTTTTTTTTTTTTTTTTTTTTTTTTTTTTTT<br>TTTTTTTTTTTTTTTTTTTTTTTTCGACAAATGCATGAAATGA<br>GAATTG	174
rs4845454_T	TTTTTTTTTTTTTTTTTTTTTTTTTTTTTTTTTTTTTTTTTTTTTT<br>TTTTTTTTTTTTTTTTTTTTTTTTTTCGACAAATGCATGAAAT<br>GAGAATTA	176
rs4085613_modify	P-GCAGTAAAAATAAATAGGCAGTTTGTTTTTTTTTTTTTTTT<br>TTTTTTTTTTTTTTTTTTTTTTTTTTTTTTTTTTTTTT-FAM	

rs4085613_A	TTTTTTTTTTTTTTTTTTTTTTTTTTTTTTTTTTTTTTTTTTTTTT<br>TTTTTTTTTTTTTTTTTTTTTTTTTTTTTAGAAATCCTGAGTTC<br>TTAGACTTT	178
rs4085613_C	TTTTTTTTTTTTTTTTTTTTTTTTTTTTTTTTTTTTTTTTTTTTTT<br>TTTTTTTTTTTTTTTTTTTTTTTTTTTTTTTAGAAATCCTGAGT<br>TCTTAGACTTG	180
rs1886734_modify	P-AAGGAAAAGGATGGGCAAGCTCCTTTTTTTTTTTTTTTTTT<br>TTTTTTTTTTTTTTTTTTTTTTTTTTTTTTTTTTTTTT-FAM	
rs1886734_G	TTTTTTTTTTTTTTTTTTTTTTTTTTTTTTTTTTTTTTTTTTTTTT<br>TTTTTTTTTTTTTTTTTTTTTTTTTTTTTTTTGAATTAAAACAC<br>ATAATGGAGAAAC	182
rs1886734_T	TTTTTTTTTTTTTTTTTTTTTTTTTTTTTTTTTTTTTTTTTTTTTT<br>TTTTTTTTTTTTTTTTTTTTTTTTTTTTTTTTTTGAATTAAAAC<br>ACATAATGGAGAAAA	184

---

article title: Association between LCE gene polymorphisms and psoriasis vulgaris among Mongolians from Inner Mongolia

journal name: Archives of Dermatological Research

F DERMATOLOGICAL RESEARCH

author names: Li Sun[1¶*], Yuting Cao[2¶], NagongbiLige[3¶], Jianwen Han[1], Hairong[4], Sharinai[3], Baoyindeligeer[3], Wurina[5], Lizhong Li[6], Xiulan Su[7], Hongwei Cui[7], Wenchao Zhao[8], Qiqige[5], Dandan Liu[2], Zhiqiang Sun[1], Yanping Huang[1]

e-mail address of the corresponding author: sunnyhong618@163.com
